# Anti‐HMGB1 Antibody Therapy Ameliorates Depression Following Spinal Cord Injury in Rats by Inhibiting Ferroptosis

**DOI:** 10.1111/jcmm.71255

**Published:** 2026-06-23

**Authors:** Zhiwu Wu, Tao Li, Qinglin Zhong, Jinshi Zhang, Yancong Yang, Jinxiang Liu, Xinyun Ye, Qiuhua Jiang, Kaiming Feng, Qianliang Huang

**Affiliations:** ^1^ Department of Neurosurgery Ganzhou Hospital‐Nanfang Hospital, Southern Medical University (Ganzhou People's Hospital) Ganzhou China

**Keywords:** depression, ferroptosis, HMGB1, neuron, spinal cord injury

## Abstract

Depression following spinal cord injury (D‐SCI) refers to a depressive state that occurs in an individual after a major spinal cord injury (SCI), characterized mainly by low mood and reduced interest. This study aims to investigate the regulatory role of anti‐HMGB1 antibody in the depressive‐like behaviour of D‐SCI rats and to explore its underlying mechanisms. A depression model was established in rats 5 weeks after SCI. The expression of HMGB1 and ferroptosis markers (MDA, GSH and iron ion deposition) in the hippocampus were examined in both the sham group and the D‐SCI group. Subsequently, D‐SCI rats were treated with an anti‐HMGB1 antibody, and the depression‐like behaviours of each group were assessed using open field and sucrose preference tests. Ferroptosis levels in the hippocampus, as well as the expression of ferroptosis‐related proteins (ACSL4, SLC7A11 and GPX4), were also investigated. The co‐localization of HMGB1 and NeuN in the rat hippocampus was detected by immunofluorescence double staining. Furthermore, at the cellular level, the effect of the anti‐HMGB1 antibody on Erastin‐induced ferroptosis in rat hippocampal neurons was analysed. The results indicated that compared to the sham group, the levels of HMGB1 and ferroptosis in the hippocampus of rats in the D‐SCI group were significantly elevated. Administering anti‐HMGB1 antibody to D‐SCI rats could significantly augment their activity distance, movement speed and sucrose preference rate, while also suppressing the ferroptosis level and the expression of ferroptosis‐related proteins in the hippocampus. Moreover, HMGB1 and NeuN were co‐expressed in the rat hippocampus. The results from primary rat hippocampal neurons indicated that anti‐HMGB1 antibody could inhibit erastin‐induced ferroptosis in rat hippocampal neurons. Taken together, anti‐HMGB1 antibody therapy can ameliorate depressive behaviour in D‐SCI rats; the possible mechanism may involve the inhibition of ferroptosis in hippocampal neurons.

## 1 Introduction

Spinal cord injury (SCI) is a chronic condition typically induced by spinal trauma, leading to damage to the spinal cord or nerves. It not only impacts the physical and mental well‐being of patients but also places a substantial burden on families and society [1, 2]. In clinical settings, it has been observed that patients following SCI frequently experience complications such as cognitive impairment, anxiety disorders and depression [3, 4, 5]. Although the statistics from various studies differ, the prevalence of depression and anxiety disorders among patients with chronic SCI is continuously rising [6, 7]. Basic research demonstrates that in animal models, depression‐like behaviours manifest after SCI [8]. Currently, there is relatively limited attention given to depression following spinal cord injury (D‐SCI), and the specific mechanism remains incompletely understood. In‐depth research on the pathogenesis of D‐SCI holds significant clinical and research value.

Ferroptosis is a type of programmed cell death that is related to iron‐dependent lipid peroxidation metabolism. It regulates cell death through the NADPH/H+ pathway, polyunsaturated fatty acid metabolism and glutamine catabolic pathway [9, 10, 11]. Previous studies have shown that ferroptosis is involved in the pathogenesis of D‐SCI. According to research by Zhou and colleagues [12], electroacupuncture treatment was shown to reduce depressive‐like behaviours in rats suffering from SCI through the suppression of neuronal ferroptosis. In a separate study, Li et al. demonstrated that hyperbaric oxygen therapy could mitigate iron‐induced neuronal death following spinal cord injury by activating the Nrf2/GPX4 signalling pathway [13].

High‐mobility group box 1 (HMGB1) is a non‐histone protein found on chromosomes. It was named because of its high electrophoretic migration rate in polyacrylamide gels [14]. HMGB1 has various physiological and pathological functions, and its localization varies depending on the function [15]. Studies have shown that HMGB1 is an important regulatory factor in the formation of depression [16]. Oral administration of the HMGB1 inhibitor glycyrrhizic acid can improve the depressive‐like behaviours of mice with neuropathic pain [17]. Furthermore, different studies have shown that HMGB1 can regulate ferroptosis in various types of cells, including neurons. The research conducted by Zhao et al. indicates that inhibiting the expression of HMGB1 in renal tubular epithelial cells can prevent epithelial cell ferroptosis induced by renal ischemia/reperfusion [18]. Wu et al.'s research on mesangial cells also revealed that HMGB1 can inhibit iron death in mesangial cells induced by high glucose [19]. Zhu et al. discovered in the model of neonatal hypoxic–ischemic brain damage (HIBD) that HMGB1 can inhibit ferroptosis of neurons in HIBD [20]. Nevertheless, to date, few studies have reported the regulatory role of anti‐HMGB1 antibody in neuronal ferroptosis after D‐SCI in rats or elucidated its underlying molecular mechanisms. Therefore, the present study aimed to investigate the regulatory role of anti‐HMGB1 in depressive behaviour in D‐SCI rats and to explore its underlying molecular mechanisms.

## 2 Materials and Methods

### 2.1 Materials

The reagents used in this study were sourced from multiple commercial suppliers. Solaibao Technology Co. Ltd. provided the HE staining kit and the Prussian blue iron staining kit. Antibodies targeting β‐Actin, HMGB1, ACSL4, GPX4, SLC7A11, NeuN and NSE were acquired from Abcam. Santa Cruz was the supplier for the anti‐HMGB1 antibody and anti‐IgG antibody. MedChemExpress LLC was the source for the FerroOrange probe and the compound erastin. Additionally, the MDA and GSH assay kits were purchased from the Nanjing Jiancheng Bioengineering Institute, while the total iron assay kit was procured from Servicebio. Sh‐HMGB1‐1, sh‐HMGB1‐2, sh‐HMGB1‐3, sh‐NC, ACSL4 overexpression (OE‐ACSL4) lentiviral vector and OE‐NC were obtained from General Biotechnology (Anhui) Co. Ltd.

### 2.2 Animals

Eighty‐seven specific pathogen free (SPF) female Sprague Dawley (SD) rats (78 were 6–8 weeks old and 9 were 17–18 days old) were purchased from Henan Skabes Biotechnology Co. Ltd. (licence No. SCXK (Yu) 2025‐0005). All experiments followed ethical guidelines approved by the Ganzhou People's Hospital (No. D2025‐010‐01). Rats were randomly assigned to different experimental groups using a computer‐generated random number sequence.

### 2.3 Establishment of the Rat D‐SCI Model

After 1 week of adaptive feeding, the model was established in rats. First, the surgical area on the back of the rats was disinfected and shaved. A 2.5 cm long incision was made centred at T10 along the midline of the spine, and the muscles were bluntly separated to fully expose T9‐T11. The spinous process and lamina of the T10 segment were removed using bone‐nipping forceps. The T10 segment spinal cord was hit with a free‐fall impact device. If there was rapid local congestion and swelling after the injury, and both hind limbs twitched and the tail swayed, it was considered that the spinal cord injury model had been successfully established. In this study, D‐SCI was operationally defined as the manifestation of validated depression‐like behaviours during the chronic phase of spinal cord injury. Based on previous studies [8, 21], the 5‐week post‐SCI time point was chosen for assessment, as it represents a stable period where injury‐induced neuropathic and psychological comorbidities robustly emerge.

To avoid the confounding effects of SCI‐induced locomotor deficits on behavioural evaluation, we primarily utilized the sucrose preference test, a gold‐standard and motor‐independent assay for anhedonia (a core symptom of depression). Specifically, our criteria and baseline values for selective inclusion were defined as follows: Screening process: At 4 weeks post‐SCI, all injured animals underwent the sucrose preference test. Cut‐off baseline value: Based on our pre‐experimental and Sham group data, animals were considered to exhibit a clear depressive‐like phenotype if their sucrose preference dropped below 65%. Group assignment: Only the animals meeting this criterion were definitively included in the D‐SCI group for subsequent experiments. Animals that maintained a sucrose preference above this threshold were considered non‐depressed/resilient and were excluded from the D‐SCI cohort. The results were shown in Table S1.

### 2.4 Experimental Design

First, the rats were divided into two groups: sham, D‐SCI (*n* = 9). Rats in both the D‐SCI group and the sham group were euthanized to collect hippocampal tissues for subsequent analysis. Then, the rats were divided into five groups: sham, model, model+PBS, model+anti‐IgG, model+anti‐HMGB1 (*n* = 12). The D‐SCI model in rats was established according to the aforementioned method. The model+anti‐HMGB1 group of rats received nasal administration (anti‐HMGB1, 200 μg/kg) 5 weeks post‐SCI [22], while those in the model+anti‐IgG and model+PBS groups were administered an equivalent volume of IgG and phosphate‐buffered saline (PBS), respectively. The sample size was determined a priori by power analysis based on our preliminary experimental data (power = 0.8, alpha = 0.05). All subsequent behavioural tests, histological assessments and molecular analyses were conducted by independent investigators who were blinded to the animal group assignments to ensure objective evaluation.

### 2.5 Open Field Test

Prepare a large open‐topped box with a closed bottom. Insert the rats into the box from the centre. Initiate the behavioural software and document the rats' activities within the box for a duration of 15 min. Monitor the total movement distance of the rats, the time they spend in the central area of the box, and their average speed. Once the experiment for each rat is concluded, remove the rat, clean the box, spray alcohol to eliminate the odour, and then commence the next experiment. Assess the rats' autonomous movement behaviour and anxiety behaviour by recording the total distance in zone, the number in the centre zone and the average speed.

### 2.6 Sucrose Preference Test

Before the experiment began, the rats were given two bottles of 1% sucrose solution to drink for 24 h. After the rats were deprived of water but not food for 24 h, the rat cages were randomly placed with equal amounts of sucrose solution and sterile water. The rats were allowed to freely drink 1% sucrose solution and an equal amount of clean water for 6 h. Sucrose preference rate = sucrose water consumption/(water consumption + sucrose water consumption) × 100%.

### 2.7 HE Staining

The rat brain tissue was fixed with 10% neutral formalin, dehydrated and embedded in paraffin. It was then cut into 4 μm tissue sections. The sections were stained with haematoxylin, rinsed with tap water and then differentiated with 1% hydrochloric acid alcohol. After that, they were stained with eosin and finally dehydrated with gradient alcohol, cleared with xylene and sealed with neutral resin. The sections were observed and photographed under an optical microscope.

### 2.8 Reverse Transcription Quantitative PCR (RT‐qPCR)

The samples were collected and total RNA was isolated using a Total RNA Miniprep Kit. Subsequently, cDNA synthesis was carried out following the instructions provided by the manufacturer. A RT‐qPCR analysis was conducted under the following conditions: Initial denaturation at 95°C for 5 min, followed by denaturation at 95°C for 10 s, annealing at 60°C for 30 s, extension from 65°C to 95°C with a temperature increment of 0.5°C every 5 s. Following completion of the reaction, the average cycle threshold (Ct) values were determined for each gene as well as for the reference gene. The relative expression levels of genes were evaluated using the widely accepted method known of 2^−ΔΔCT^. The sequences of the primers are shown in Table 1.

**TABLE 1:** Primer sequences.

Gene symbol	Forward primer	Reverse primer
HMGB1	AAAGGAGATCCTAAGAAGCCGA	TCATAACGAGCCTTGTCAGCC
ACSL4	TCAAGCATTCCTCCAAGTAGACC	CAGCCGTAGGTAAAGCAGGAG
SLC7A11	GCTGGCTGGTTTTACCTCAACT	CCTCGGCGCTAATGGTTGTA
GPX4	AGGCAGGAGCCAGGAAGTAATC	ACCACGCAGCCGTTCTTATC
GAPDH	CTGGAGAAACCTGCCAAGTATG	GGTGGAAGAATGGGAGTTGCT

### 2.9 Western Blotting

Total protein was extracted and the content was assessed using a bicinchoninc assay protein assay kit. Subsequently, the protein (30 μg) was separated by 10% SDS‐PAGE and transferred onto a polyvinylidene difluoride membrane. The membranes used for the target protein (and β‐actin) were blocked with 5% skimmed milk at 25°C for 1 h. The corresponding membranes were incubated with the following primary antibodies: HMGB1 (1:1000), ACSL4 (1:1000), GPX4 (1:1000) and SLC7A11 (1:1000), followed by incubation with a secondary antibody for 1 h. Finally, the protein bands were assessed by an enhanced chemiluminescence (ECL)‐detecting kit, and β‐actin was used as a loading control.

### 2.10 Detection MDA and GSH Levels

Rat hippocampus or cell samples were collected, and the levels of MDA and GSH were determined in accordance with the protocols outlined in the respective kit instructions.

### 2.11 Iron Content Assay

Rat hippocampal tissues were collected, and the iron ion content in the tissues from each experimental group was measured according to the protocol provided in the total iron ion detection kit instructions.

### 2.12 Prussian Blue Staining

The rat hippocampus was first fixed and embedded in paraffin, followed by sectioning. The tissue sections were then deparaffinized and rehydrated through a graded ethanol series to distilled water. Subsequently, the sections were incubated in a freshly prepared staining solution containing equal volumes of 2% potassium ferrocyanide and 2% hydrochloric acid for 30 min, followed by two washes with distilled water. Thereafter, 3,3′‐Diaminobenzidine (DAB) chromogenic solution was applied to the sections for approximately 10 min. The reaction was terminated by removing the DAB solution, after which the sections were rinsed once with 0.01 mol/L PBS and three times with distilled water. Nuclei were counterstained with haematoxylin. Finally, the sections were dehydrated through an ascending alcohol series, cleared in xylene, mounted with neutral balsam and examined under a light microscope for imaging. Following staining, iron‐rich regions in the tissue appeared brown, while nuclei were stained light blue.

### 2.13 Isolation of Primary Hippocampal Neuron

Utilize 9 fetal rats at 17–18 days of gestation for the isolation of hippocampal neurons. Administer deep anaesthesia to the young rats, disinfect them and subsequently decapitate them. Conduct the operation on ice, excise the epidermis and skull to fully expose the brain tissue. Isolate the hippocampi, wash them twice with D‐Hank's solution, then collect them and place them in a 5 mL Eppendorf (EP) tube. Employ ophthalmic scissors to mince them into pieces of approximately 1 mm^3^. Add 0.125% trypsin to the centrifuge tube and incubate it in a 37°C incubator for 15 min, with intermittent shaking. Add DMEM/F12 medium containing 10% fetal bovine serum to the centrifuge tube to terminate the digestion. After dispersing the tissue, pass it through a sieve (200‐mesh sieve), collect the filtrate, centrifuge at 1000 rpm for 10 min, resuspend the cell sediment in complete medium and inoculate the cell suspension at a density of 3 × 10^6^/mL onto pre‐coated polylysine T75 cell culture flasks. Approximately 10 mL of complete medium is added to each flask, and the flasks are placed in a 37°C, 5% CO_2_ incubator for cultivation.

### 2.14 Cell Culture and Treatment

Primary rat hippocampal neurons were cultured in a specialized medium (DMEM/F12 medium supplemented with 1% glutamine, 2% B27 and 1% double antibody). The hippocampal neurons were treated with 10 μM erastin for 8 h to simulate the in vitro cell ferroptosis model. In the Erastin+anti‐HMGB1 group, the cells were treated with 1 μg/mL anti‐HMGB1 antibody [22] for 30 min after the model was established. While in the Erastin+PBS group, the cells were treated with the same dose of PBS.

### 2.15 FerroOrange Staining

Neurons were seeded into fluorescent culture dishes and incubated overnight under controlled conditions at 37°C in a humidified atmosphere with 5% CO_2_. Following incubation, the supernatant was carefully removed, and cells were rinsed three times with serum‐free medium to ensure removal of residual components. Subsequently, 1 μM FerroOrange working solution was added to the cells, which were then incubated in the dark at 37°C for 30 min. Fluorescence imaging was performed using a fluorescence microscope equipped with excitation at 543 nm and emission detection at 580 nm.

### 2.16 Transmission Electron Microscopy (TEM)

The cells were fixed in electron microscope fixation solution for about 30 min. The cells were double stained with 3% uranium acetate and lead citrate. The ultrastructure of the cells was observed by transmission electron microscope and photographed for preservation.

### 2.17 Co‐Immunoprecipitation (Co‐IP) Assay

Neuronal cell samples were collected and lysed, followed by centrifugation to obtain the supernatant. One microgram of the corresponding antibody (HMGB1 or IgG antibody) was added to the cell lysate and incubated at 4°C overnight with gentle agitation. 10 μL protein A/G agarose beads were prepared by washing three times with an appropriate volume of lysis buffer via centrifugation at 3000 rpm for 3 min per wash. The pre‐washed protein A/G agarose beads (10 μL) were then added to the lysate that had been incubated with the antibody, and the mixture was incubated at 4°C for 4 h under gentle shaking to facilitate antibody‐bead conjugation. Following incubation, the sample was centrifuged at 3000 rpm for 3 min at 4°C to pellet the agarose beads. The supernatant was carefully removed, and the beads were washed 3–4 times with 1 mL of lysis buffer. Finally, 15 μL of 2× SDS loading buffer was added to the beads, and the samples were boiled for 5 min to elute bound proteins. Subsequent analysis was performed using SDS‐PAGE and Western blotting to detect the immunoprecipitated proteins.

### 2.18 Statistical Analyses

SPSS 22.0 (IBM Corp.) was applied for statistical analysis and the experimental data were expressed as mean ± standard deviation (*x* ± *s*). The normality of the data distribution was first verified using the Shapiro–Wilk test. For normally distributed data, statistical differences between two groups were analysed using an unpaired two‐tailed Student's *t*‐test, while comparisons among three or more groups were performed using a one‐way analysis of variance (ANOVA) followed by Tukey's post hoc test. *p* < 0.05 was considered to indicate a statistically significant difference.

## 3 Results

### 3.1 Upregulation of HMGB1 and Ferroptosis Was Observed in the Hippocampal Tissues of Rats With D‐SCI

**FIGURE 1:**
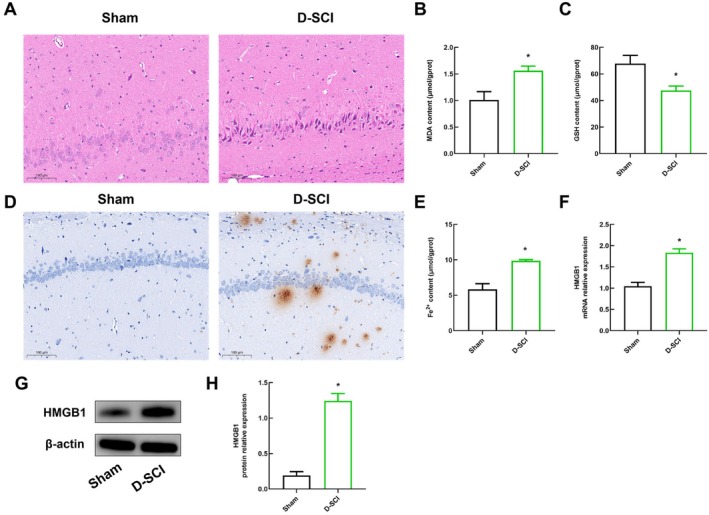
The levels of HMGB1 and ferroptosis significantly elevated in the hippocampal tissue of D‐SCI rats. (A) HE staining (*n* = 3). (B–C) The concentrations of (B) MDA (*n* = 3) and (C) GSH (*n* = 3) in rat hippocampal tissue were measured using biochemical assay kits. (D) Prussian blue staining was employed to assess iron deposition in hippocampal tissue of rats (*n* = 3). (E) The total iron assay kit was employed to measure the iron content in rat hippocampal tissue (*n* = 3). (F–H) The mRNA and protein expression levels of HMGB1 in rat hippocampal tissue were detected by (F) RT‐qPCR (*n* = 3) and (G–H) western blotting (*n* = 3). The data are presented as mean ± SD. *p < 0.05 versus Sham.

Firstly, we used HE staining to observe the pathological differences in the hippocampal tissues between the sham group and the D‐SCI group of rats (Figure 1A). The results show that the neuronal cell structures of the rats in the sham group were intact and well arranged. In contrast, the neuronal cells of the rats in the D‐SCI group exhibited neuronal shrinkage, partial nuclear fragmentation and blurred nucleoli. Then, the concentrations of MDA (Figure 1B) and GSH (Figure 1C) were quantified in the hippocampus across distinct rat groups. Compared with the sham group, the MDA level was significantly elevated in the D‐SCI rats, while the GSH level was markedly reduced. Furthermore, prussian blue staining was employed to detect iron accumulation in the rat hippocampus across various groups (Figure 1D). No iron deposition was observed in the sham group, whereas obvious iron deposition was observed in the D‐SCI group of rats. As shown in Figure 1E, we further used a total iron ion detection kit to examine the differences of iron ion levels in different groups. Compared with the sham group, a significant elevation in iron ion levels was observed in the D‐SCI group. Afterwards, the mRNA and protein expression levels of HMGB1 in different rat hippocampi were detected by RT‐qPCR and western blotting, respectively (Figure 1F–H). Compared with the sham group, the mRNA and protein expression levels of HMGB1 were significantly increased in the D‐SCI group.

### 3.2 Treatment With Anti‐HMGB1 Antibody Enhanced Depression‐Like Behaviours in Rats With D‐SCI

**FIGURE 2:**
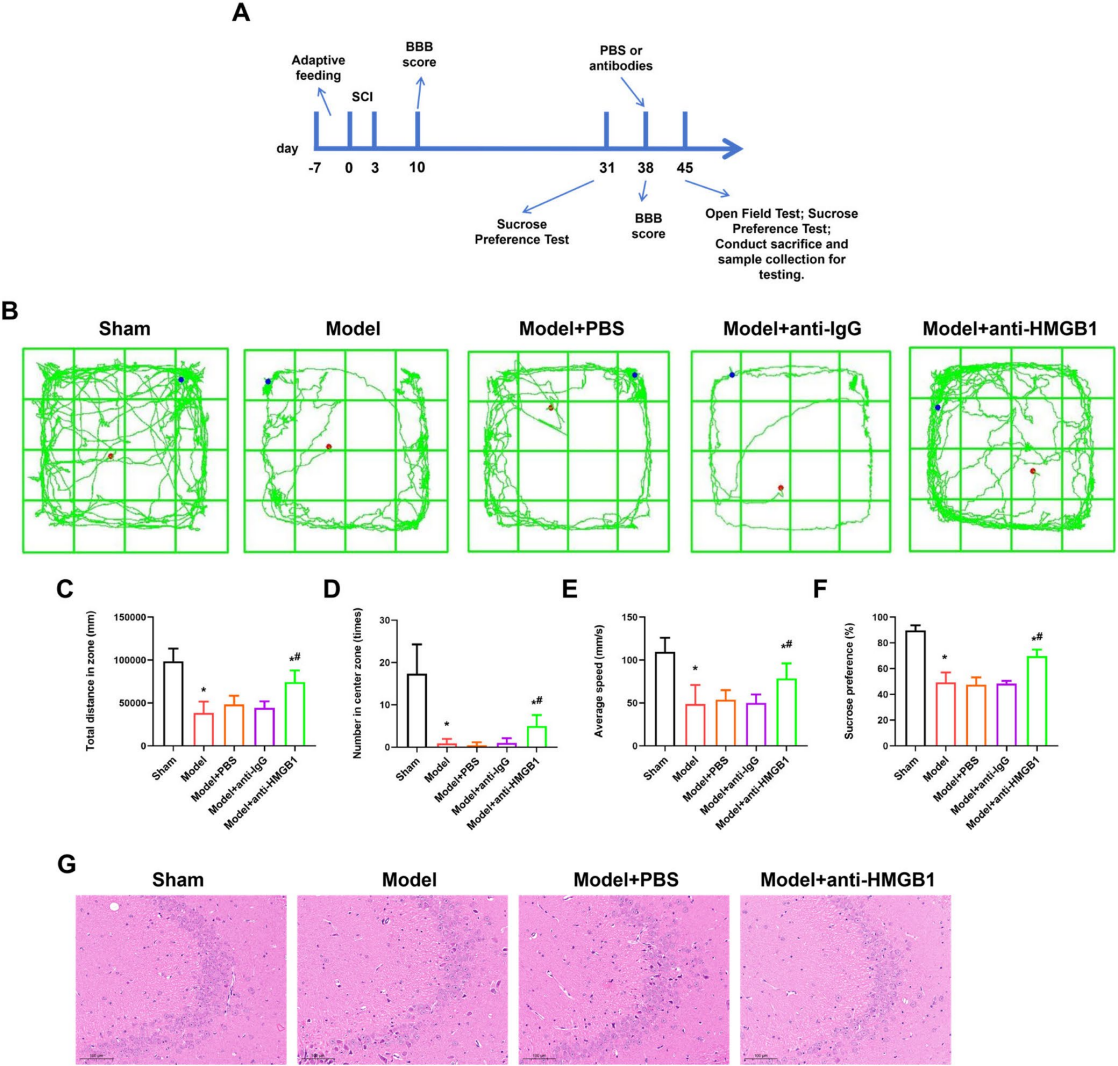
Effect of anti‐HMGB1 antibody on the depression‐like behaviours and brain pathology of the D‐SCI rats. (A) A workflow of animal part experiments. (B–E) Open field test (*n* = 12). (F) Sucrose preference (*n* = 12). (G) HE staining (*n* = 3). The data are presented as mean ± SD. *p < 0.05 versus Sham; ^#^ *p* < 0.05 versus Model+PBS.

Firstly, we used the open field test to measure the spontaneous activity levels, exploration behaviours and anxiety‐like states of the rats in each group (Figure 2A,B). The results showed that compared with the sham group, the total distance in zone, the number in the centre zone and the average speed of the rats in the model group were all significantly reduced. In addition, total distance in zone, the number in the centre zone and the average speed of the rats in the model+anti‐HMGB1 group were significantly enhanced compared with the model+PBS group and the model+anti‐IgG group (Figure 2C–E). Subsequently, we conducted a sucrose preference experiment on each group of rats (Figure 2F). In comparison with the rats in the control group, the sucrose preference rate of the rats in the model group was notably lower. Nevertheless, intervention with anti‐HMGB1 antibody significantly increased the sucrose preference rate in the model rats. Furthermore, we used HE staining to observe the pathological differences in the hippocampus of different rats (Figure 2G). Compared with the model+PBS group of rats, the hippocampal neuronal cell structures in the model+anti‐HMGB1 group were significantly restored, with a reduction in nuclear rupture and blurred nucleoli.

To exclude the confounding effects of SCI‐induced motor deficits on mood‐related behavioural readouts, we evaluated hindlimb motor function using the BBB scale. As shown in Figure S1, both model+PBS group and model+anti‐HMGB1 group exhibited severe motor impairment with no significant difference in locomotor scores at 5 weeks post‐injury. This standard functional evaluation confirms that the injury severity was uniform. Consequently, the improvements observed in the sucrose preference test and specific exploratory parameters in the open field test in the model+anti‐HMGB1 group can be more reliably attributed to an amelioration of depression‐like behaviour (anhedonia and decreased exploration) rather than an artefact of differential motor recovery.

### 3.3 Treatment With Anti‐HMGB1 Antibody Alleviated Ferroptosis in the Hippocampus of D‐SCI Rats

**FIGURE 3:**
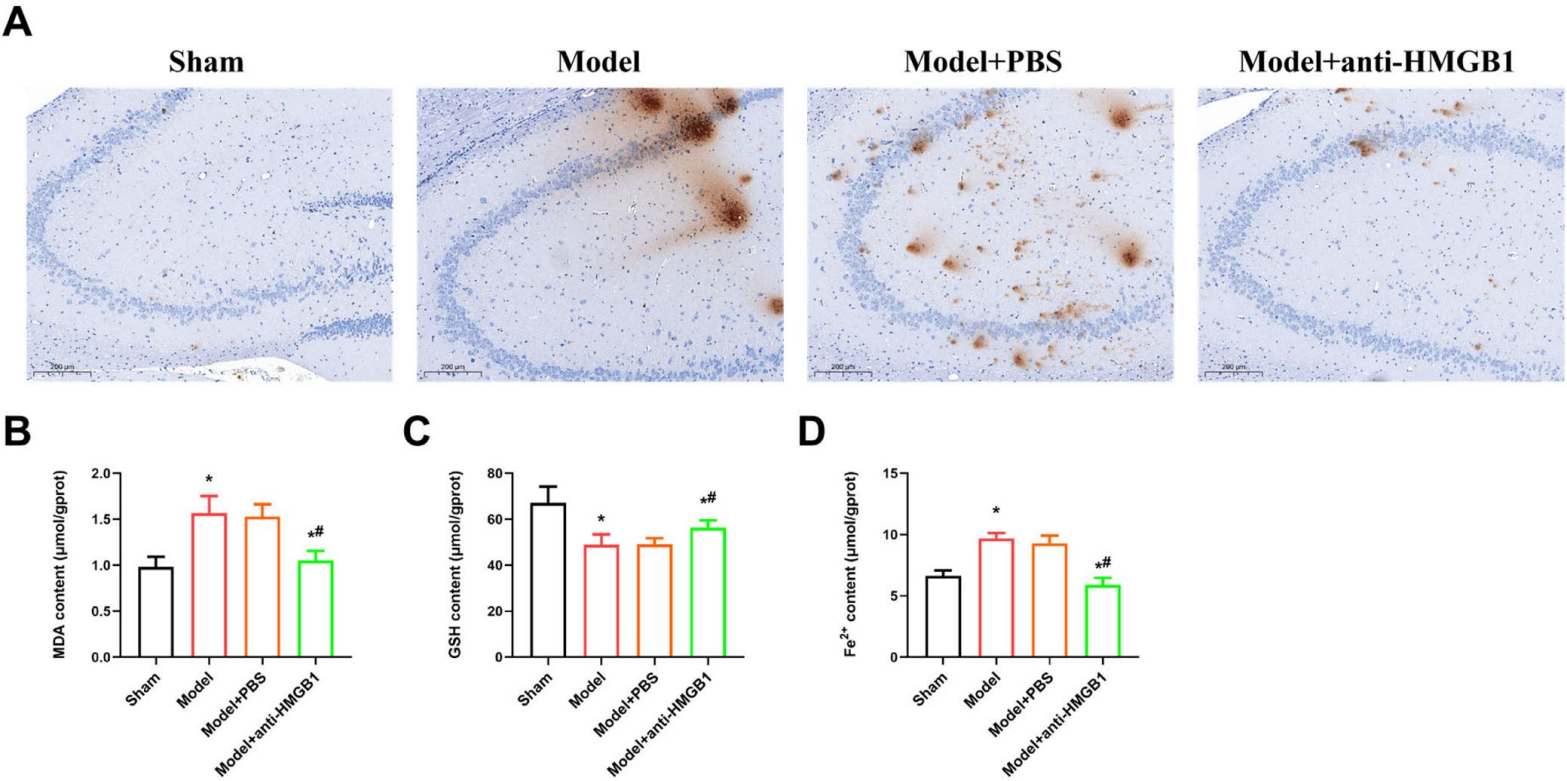
Effect of anti‐HMGB1 antibody on the ferroptosis in the hippocampus tissue of D‐SCI rats. (A) Prussian blue staining was employed to assess iron deposition in hippocampal tissue of rats (*n* = 3). (B, C) The concentrations of (B) MDA (*n* = 3) and (C) GSH (*n* = 3) in rat hippocampal tissue were measured using biochemical assay kits. (D) The total iron assay kit was employed to measure the iron content in rat hippocampal tissue (*n* = 3). The data are presented as mean ± SD. *p < 0.05 versus Sham; ^#^ *p* < 0.05 versus Model+PBS.

As shown in Figure 3A, we used prussian blue staining to detect iron accumulation in the rat hippocampus across various groups. Compared with the model+PBS group of rats, the iron deposition in the hippocampus of the model+anti‐HMGB1 group was reduced. Then, the concentrations of MDA (Figure 3B) and GSH (Figure 3C) were quantified in the hippocampus across distinct rat groups. Compared with the model+PBS group, the MDA level was significantly decreased in the model+anti‐HMGB1 group, while the GSH level was markedly increased. Subsequently, we used a total iron ion detection kit to examine the differences in iron ion levels in different groups (Figure 3D). Compared with the model+PBS group, a significant reduction in iron ion levels was observed in the model+anti‐HMGB1 group.

**FIGURE 4:**
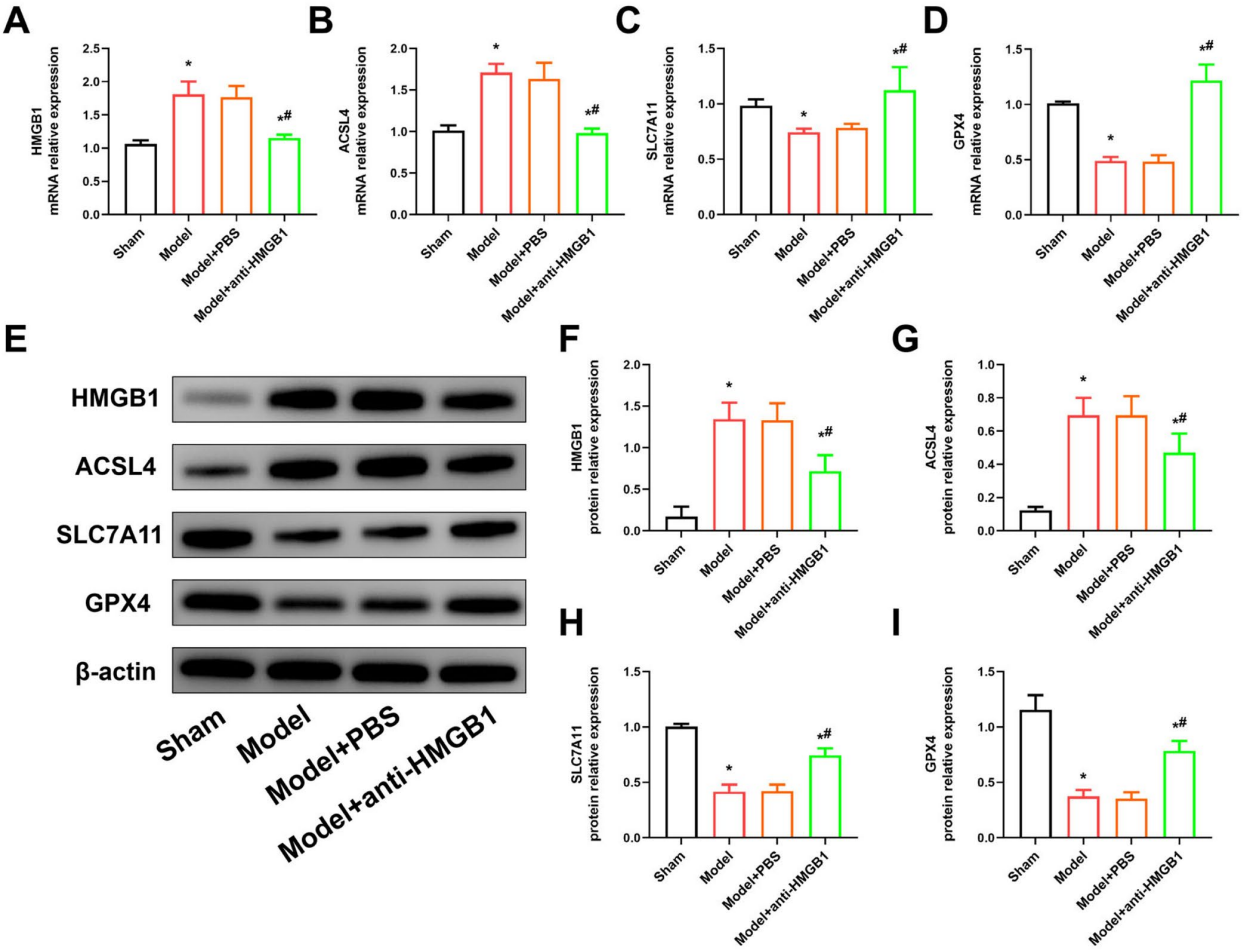
Effect of anti‐HMGB1 antibody on the expression of HMGB1, ACSL4, GPX4 and SLC7A11 in the hippocampus tissue of D‐SCI rats. (A–D) The mRNA expression levels of HMGB1, ACSL4, GPX4 and SLC7A11 in hippocampal tissue of different rats were detected by RT‐qPCR (*n* = 3). (E–I) The protein expression levels of HMGB1, ACSL4, GPX4 and SLC7A11 in hippocampal tissue of different rats were detected by western blotting (*n* = 3). The data are presented as mean ± SD. *p < 0.05 versus Sham; ^#^ *p* < 0.05 versus Model+PBS.

To further verify the effect of anti‐HMGB1 antibody on ferroptosis in rat hippocampus, we used RT‐qPCR and western blotting to assess the mRNA and protein expression levels of HMGB1, ACSL4, GPX4 and SLC7A11 in the hippocampus of different rats (Figure 4A–I). Compared with the model+PBS group, the mRNA and protein expression levels of HMGB1 were significantly lowered in the model+anti‐HMGB1 group. Besides, in comparison with the sham group, the mRNA and protein expressions of ACSL4 in the hippocampus of the model group rats exhibited a significant increase. Conversely, the mRNA and protein expressions of SLC7A11 and GPX4 showed a significant decrease. Moreover, when compared with the model+PBS group rats, the mRNA and protein expressions of ACSL4 in the hippocampus of the model+anti‐HMGB1 group rats significantly decreased, whereas the mRNA and protein expressions of SLC7A11 and GPX4 significantly increased. These results indicated that treatment with anti‐HMGB1 antibody can alleviate ferroptosis in the hippocampus of D‐SCI rats.

### 3.4 Anti‐HMGB1 Antibody Inhibited Erastin‐Induced Ferroptosis in Rat Hippocampal Neurons

**FIGURE 5:**
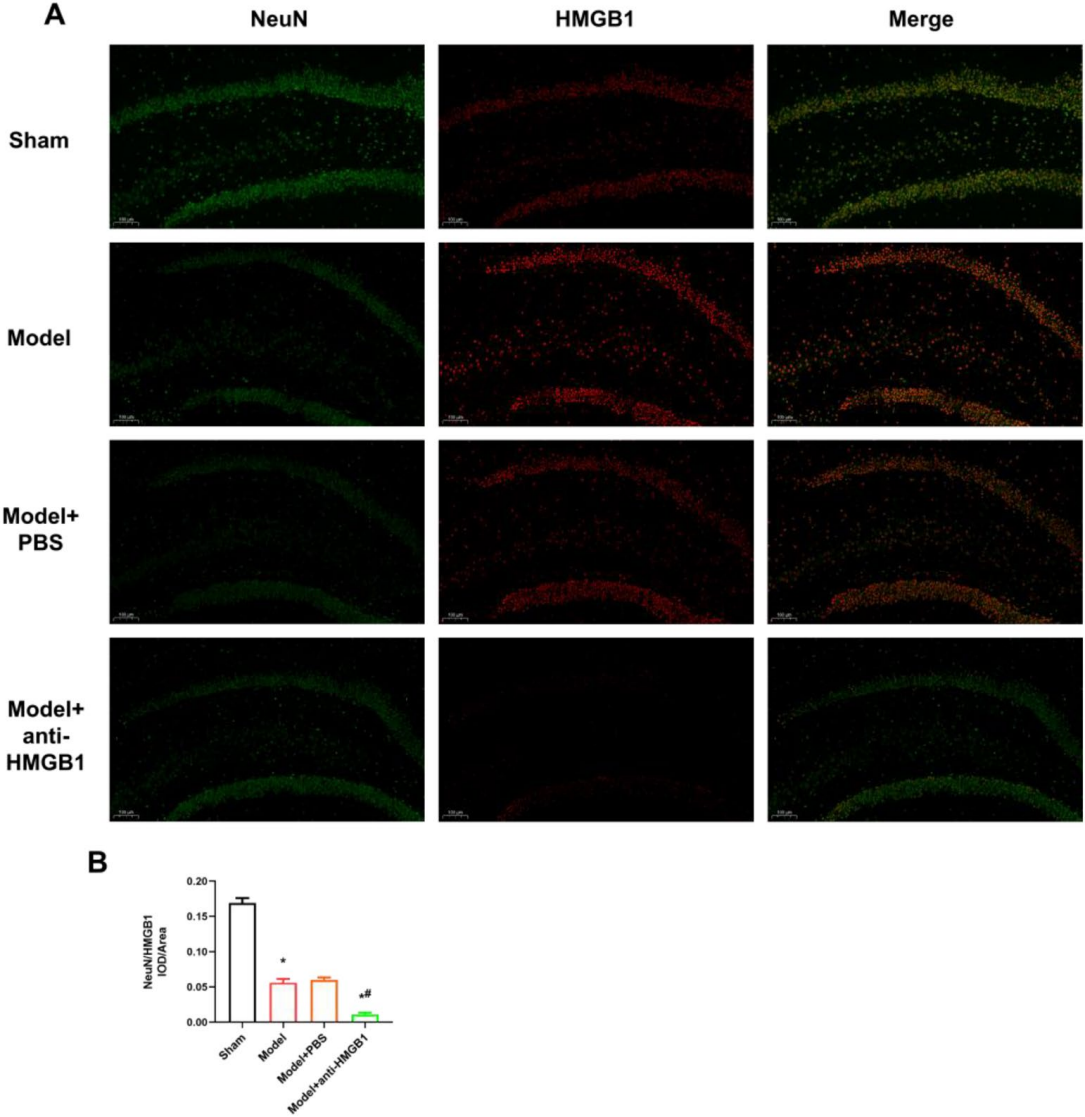
(A, B) Immunofluorescence double staining was used to detect the co‐expression of NeuN and HMGB1 in the brain tissue of rats from different groups (*n* = 3). The data are presented as mean ± SD. *p < 0.05 versus Sham; #*p* < 0.05 versus Model+PBS.

As shown in Figure 5A,B, we used immunofluorescence double staining to detect the co‐expression of HMGB1 and NeuN in different rat hippocampi. The results also showed that HMGB1 expression was significantly higher in the hippocampus of the model group rats compared with the sham group. Moreover, HMGB1 and NeuN were co‐localized in the hippocampal tissues of the rats.

**FIGURE 6:**
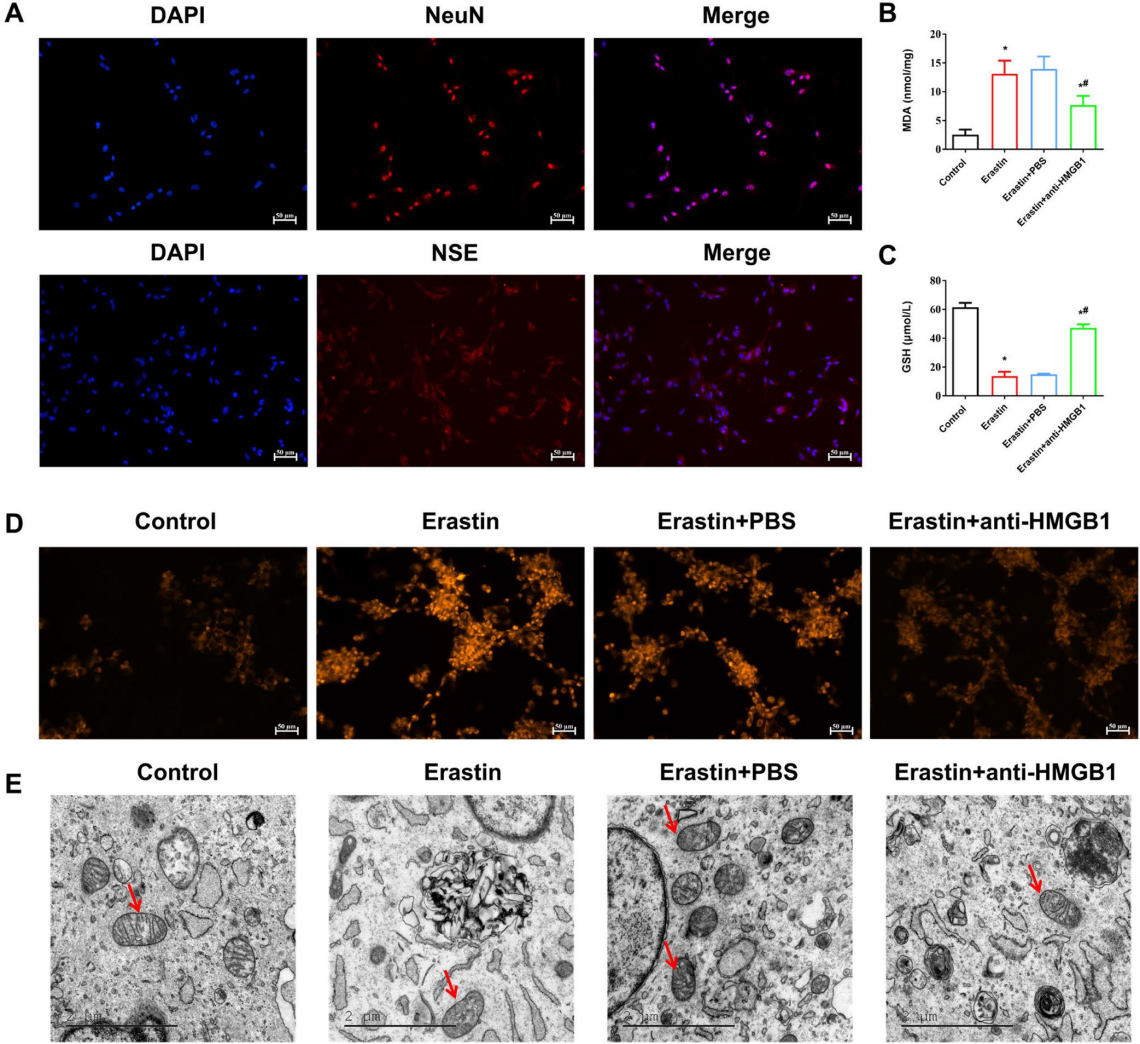
Anti‐HMGB1 antibody inhibited ferroptosis in rat hippocampal neurons. (A) NeuN and NSE were detected via immunofluorescence staining to identify rat hippocampal neurons (*n* = 3). (B, C) The concentrations of MDA and GSH in neurons of different treatments were measured using biochemical assay kits (*n* = 3). (D) The FerroOrange probes were used to detect the ferrous ion levels in neurons across different treatment groups (*n* = 3). (E) TEM assay was used to detect the microstructure of neurons in different treatment groups (*n* = 3). The data are presented as mean ± SD. *p < 0.05 versus Control; ^#^ *p* < 0.05 versus Erastin+PBS.

Therefore, we subsequently isolated primary rat hippocampal neurons and analysed the effect of anti‐HMGB1 antibody on ferroptosis of rat hippocampal neuron cells. Firstly, immunofluorescence was used to identify the marker proteins (NeuN and NSE) of rat hippocampal neurons (Figure 6A). The results indicated that NeuN and NSE were positively expressed in rat spinal cord neurons. Subsequently, the concentrations of MDA (Figure 6B) and GSH (Figure 6C) were measured in neurons subjected to various treatments. Relative to the control group, the Erastin‐treated group exhibited a marked elevation in MDA content and a significant reduction in GSH. In contrast, administration of anti‐HMGB1 alongside Erastin resulted in a substantial decrease in MDA and a notable increase in GSH levels when compared to the Erastin+PBS group. Furthermore, we used FerroOrange staining to observe the differences in ferrous ion levels in neurons among different treatment groups (Figure 6D). In comparison to the control group, neurons in the Erastin‐treated group showed an increase in ferrous ion concentrations. However, administration of an anti‐HMGB1 antibody markedly lowered the ferrous ion content compared to the Erastin+PBS group. As depicted in Figure 6E, transmission electron microscopy was next used to analyse changes in neuronal microstructure in the different experimental groups. Compared with the control group, neurons in the Erastin group exhibited loss of mitochondrial cristae. In contrast, mitochondrial morphology in the Erastin+anti‐HMGB1 group was largely restored to normal relative to the Erastin+PBS group. These results indicated that anti‐HMGB1 antibody can inhibit erastin‐induced ferroptosis in rat hippocampal neurons.

### 3.5 Treatment With Anti‐HMGB1 Antibody Inhibited the Expression of ACSL4, GPX4 and SLC7A11 in Rat Hippocampal Neurons

**FIGURE 7:**
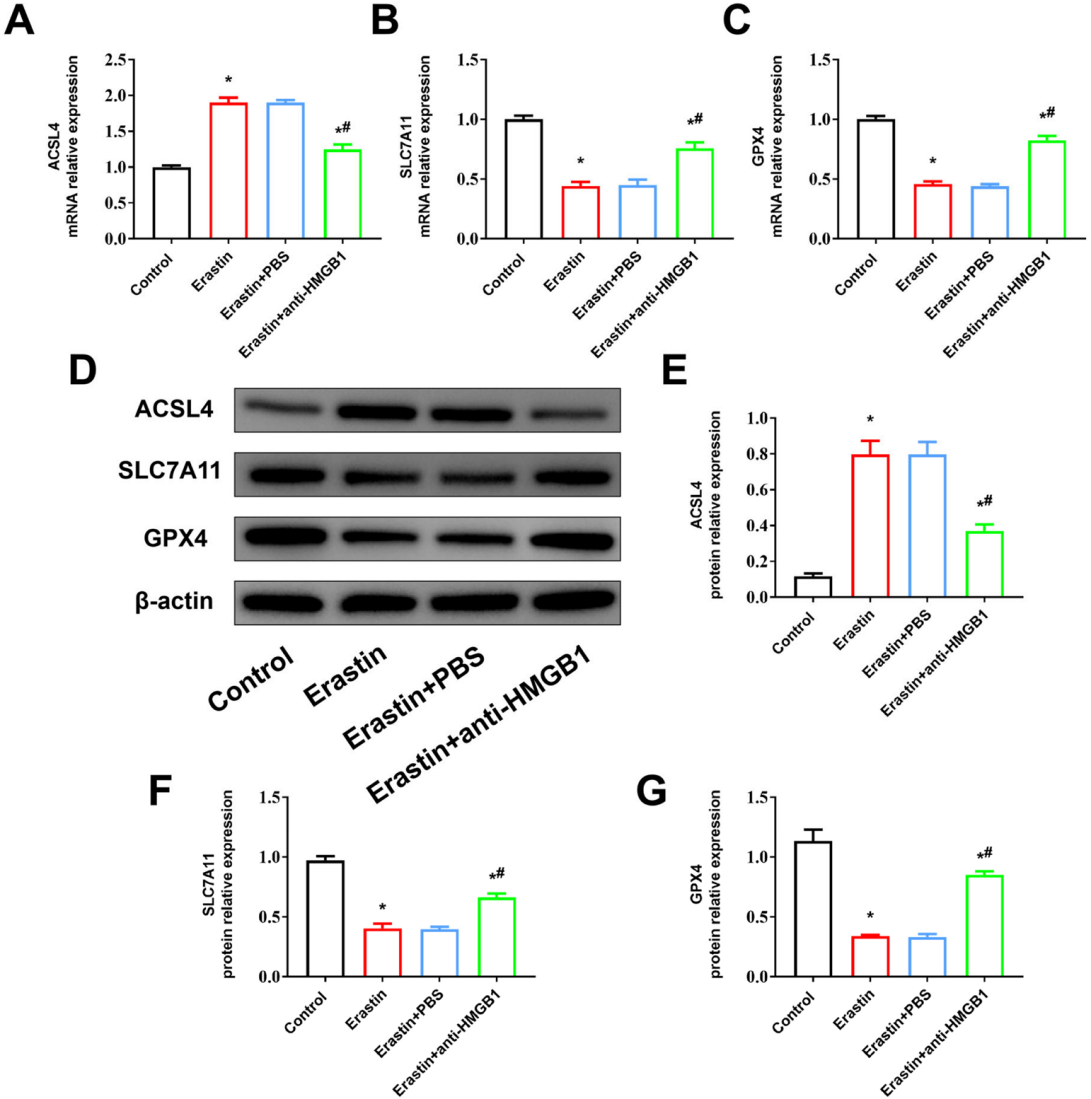
Effect of anti‐HMGB1 antibody on the expression of ACSL4, GPX4 and SLC7A11 in rat hippocampal neurons. (A–C) The mRNA expression levels of ACSL4, GPX4 and SLC7A11 in neurons of different groups were detected by RT‐qPCR (*n* = 3). (D–G) The protein expression levels of ACSL4, GPX4 and SLC7A11 in neurons of different groups were detected by western blotting (*n* = 3). The data are presented as mean ± SD. *p < 0.05 versus Control; ^#^ *p* < 0.05 versus Erastin+PBS.

As shown in Figure 7A–G, the mRNA and protein expression levels of ACSL4, SLC7A11 and GPX4 were detected by RT‐qPCR and western blotting, respectively. Relative to the control group, the erastin‐treated group exhibited a marked upregulation of ACSL4 mRNA and protein expression, alongside a significant downregulation of GPX4 and SLC7A11. Furthermore, administration of an anti‐HMGB1 antibody in erastin‐treated cells substantially reversed these effects, leading to decreased ACSL4 expression and increased GPX4 and SLC7A11 expression compared to the erastin+PBS group.

Subsequently, we transfected the HMGB1 interference vector and the ACSL4 overexpression vector into rat neuronal cells. The qPCR results indicated that the vector transfection was effective (Figure S2A,B). The results of further rescue experiments are shown in Figure S2C–E. Compared with the Erastin group, the mRNA expression level of ACSL4 was significantly decreased in the Erastin+Fer‐1 group and Erastin+shHMGB1 group, while the mRNA expression levels of GPX4 and SLC7A11 were significantly increased in the Erastin+Fer‐1 group and Erastin+shHMGB1 group. Moreover, compared with the Erastin+shHMGB1 group, the mRNA expression level of ACSL4 was significantly increased in the Erastin+shHMGB1+OE‐ACSL4 group, and the mRNA expression levels of GPX4 and SLC7A11 were significantly decreased in the Erastin+shHMGB1+OE‐ACSL4 group. To further verify the targeted interaction between HMGB1 and ACSL4 in rat hippocampal neurons, we conducted a Co‐IP experiment and the results were shown in Figure S2F. These results suggest an association between HMGB1 and ACSL4 in rat hippocampal neurons, potentially indicating co‐localization within a protein complex.

## 4 Discussion

Depression following spinal cord injury (D‐SCI) refers to an emotional disorder that occurs in individuals after suffering a major spinal cord injury. Its core characteristics include persistent low mood, reduced interest and loss of pleasure, among other clinical manifestations [23, 24]. The hippocampus, as an important structure within the limbic system of the brain, is a key brain region for the generation and regulation of emotions and motivations [25]. Numerous studies have shown that the onset of depression is closely related to the loss of hippocampal neurons and the reduction of neurogenesis in patients' brains [26, 27, 28]. Ferroptosis, as a new form of programmed cell death, has a mechanism distinct from apoptosis, necrosis and autophagy. In depression disease models, ferroptosis has been proven to be involved in neuronal damage and the progression of the disease [29, 30, 31]. The results of Jiao et al. showed that in the mouse depression model induced by chronic mild unpredictable stimulation, ferroptosis was activated in hippocampal neurons, and PEBP1‐GPX4‐mediated neuronal ferroptosis was involved in the mechanism regulation of antidepressant effects [32]. Our research results indicate that, in comparison with the sham group, iron deposition took place in the hippocampus of rats in the D‐SCI group. Moreover, the level of MDA increased significantly, whereas the level of GSH decreased significantly.

HMGB1 is a non‐histone chromosomal‐binding protein that is widely present in the nuclei of eukaryotic cells [33]. Studies have shown that HMGB1 is an important regulatory factor in the formation of depression. Xu et al. found that X‐rays in radiation‐induced brain injury would increase the expression of HMGB1 in the brain tissue of mice, thereby inducing depressive‐like behaviours in the mice [34]. Xiang et al.'s clinical study also revealed that the serum HMGB1 level of depressed adolescents was significantly higher compared to the healthy control group [35]. Our research results showed that compared to the sham group, the expression of HMGB1 in the hippocampus of rats in the D‐SCI group was also significantly increased. Subsequently, we intervened with anti‐HMGB1 antibody in D‐SCI rats. The results showed that treating D‐SCI rats with anti‐HMGB1 antibody significantly increased their activity distance, movement speed and sucrose preference rate. The research conducted by Hisaoka‐Nakashima et al. in a mouse model of neuropathic pain (NP) demonstrated that the intervention using anti‐HMGB1 antibody in NP mice could mitigate the depressive‐like behaviours of the mice [17]. This outcome is in line with our results.

The hippocampus of patients with depression often shows volume reduction and neuronal damage, and ferroptosis may be one of the mechanisms causing these pathological changes [36, 37]. Our research results also show that treating D‐SCI rats with anti‐HMGB1 antibody can inhibit the ferroptosis level in their hippocampus and the expression of ferroptosis‐related proteins. The results of in vitro cell experiments indicate that anti‐HMGB1 antibody can inhibit ferroptosis induced by Erastin in rat hippocampal neurons. The research conducted by Jia et al. on acute lung injury (SALI) in mice with sepsis showed that inhibiting HMGB1 could prevent ferroptosis in the alveolar epithelial cells of SALI mice [38]. The research results of Tian et al. on acute kidney injury (AKI) in mice also indicated that the HMGB1 inhibitor glycyrrhizic acid could inhibit ferroptosis in the renal tubular epithelial cells of AKI mice by suppressing HMGB1 [39]. These results are consistent with our own research findings.

Based on the results of the present study, it can be concluded that anti‐HMGB1 antibody therapy can ameliorate depressive behaviour in D‐SCI rats; the possible mechanism may involve the inhibition of ferroptosis in hippocampal neurons. However, the present study exhibits certain limitations. The results of this study merely demonstrate the regulatory impact of HMGB1 on ferroptosis in rat hippocampal neurons, but do not analyse the specific targets for regulating ferroptosis. In our subsequent research, we will carry out transcriptome sequencing and retrospective experiments to analyse the specific targets of HMGB1 in regulating ferroptosis in rat hippocampal neurons.

## 5 Conclusion

In summary, these studies revealed that anti‐HMGB1 antibody therapy can ameliorate depressive behaviour in D‐SCI rats. The possible mechanism may involve the inhibition of ferroptosis in hippocampal neurons. Further studies should analyse the specific targets of HMGB1 in regulating ferroptosis in rat hippocampal neurons.

## Author Contributions

**Jinshi Zhang:** writing – original draft, methodology, software, data curation, investigation. **Qinglin Zhong:** writing – original draft, methodology, software, data curation, investigation. **Tao Li:** writing – original draft, conceptualization, investigation, methodology, software. **Zhiwu Wu:** writing – original draft, conceptualization, methodology, data curation, funding acquisition, software. **Qiuhua Jiang:** writing – review and editing, conceptualization, visualization. **Xinyun Ye:** writing – review and editing, visualization, software. **Jinxiang Liu:** writing – original draft, methodology, software, data curation, investigation. **Yancong Yang:** writing – original draft, methodology, software, data curation, investigation. **Qianliang Huang:** writing – review and editing, visualization, conceptualization, formal analysis. **Kaiming Feng:** writing – review and editing, visualization, conceptualization.

## Funding

This work was supported by the Science and Technology Plan project of Ganzhou City (No. 2023NS127385), the Research Project of the Jiangxi Provincial Administration of Traditional Chinese Medicine (No. 2025022792 and No. 2024B0615) and the Natural Science Foundation of Jiangxi Province (No. 20242BAB20380).

## Ethics Statement

The animal study was approved by the Scientific Committee of the Ganzhou People's Hospital (No. D2025‐010‐01). The study was conducted in accordance with the local legislation and institutional requirements.

## Consent

All the authors have agreed to the content of the manuscript and its publication.

## Conflicts of Interest

The authors declare no conflicts of interest.

## Supporting information


**Figure S1:** The Basso, Beattie and Bresnahan (BBB) score results in different groups of rats. (A) 1 weeks post‐SCI (*n* = 12). (B) 5 weeks post‐SCI (*n* = 12). The data are presented as mean ± SD. *p < 0.05 versus Sham.
**Figure S2:** HMGB1 regulates neuronal ferroptosis through targeted modulation of ACSL4. (A) Transfection efficiency of sh‐HMGB1s in neurons was verified by RT‐qPCR. (B) Transfection efficiency of ACSL4 overexpression plasmid in neurons was verified by RT‐qPCR. (C–E) The mRNA expression levels of ACSL4, GPX4 and SLC7A11 in neurons of different groups were detected by RT‐qPCR. (F) The CO‐IP experiment was performed to investigate the specific interaction between HMGB1 and ACSL4.

**Table S1:** The sucrose preference rate of rats at 4 weeks post‐SCI.

## Data Availability

The data that support the findings of this study are available from the corresponding author upon reasonable request.
