# Supplementary material for: Anti‐HMGB1 Antibody Therapy Ameliorates Depression Following Spinal Cord Injury in Rats by Inhibiting Ferroptosis
Source: J Cell Mol Med. 2026 Jun 23;30(12):e71255. doi: 10.1111/jcmm.71255 (PMC13290662; doi:10.1111/jcmm.71255)

**Figure legends**

**Figure S1.** The Basso, Beattie, and Bresnahan (BBB) score results in different groups of rats. (**A**) 1 weeks post-SCI (n=12). (**B**) 5 weeks post-SCI (n=12). The data are presented as mean ± SD. *P<0.05 vs. Sham.

**Figure S2.** HMGB1 regulates neuronal ferroptosis through targeted modulation of ACSL4. (**A**) Transfection efficiency of sh-HMGB1s in neurons was verified by RT-qPCR. (**B**) Transfection efficiency of ACSL4 overexpression plasmid in neurons was verified by RT-qPCR. (**C-E**) The mRNA expression levels of ACSL4, GPX4 and SLC7A11 in neurons of different groups were detected by RT-qPCR. (**F**) The CO-IP experiment was performed to investigate the specific interaction between HMGB1 and ACSL4. The data are presented as mean ± SD. *P<0.05 vs. sh-NC, OE-NC or Control; ^#^P<0.05 vs. Erastin; ^@^P<0.05 vs. Erastin+sh-HMGB1. n=3.

**Fig. S1**


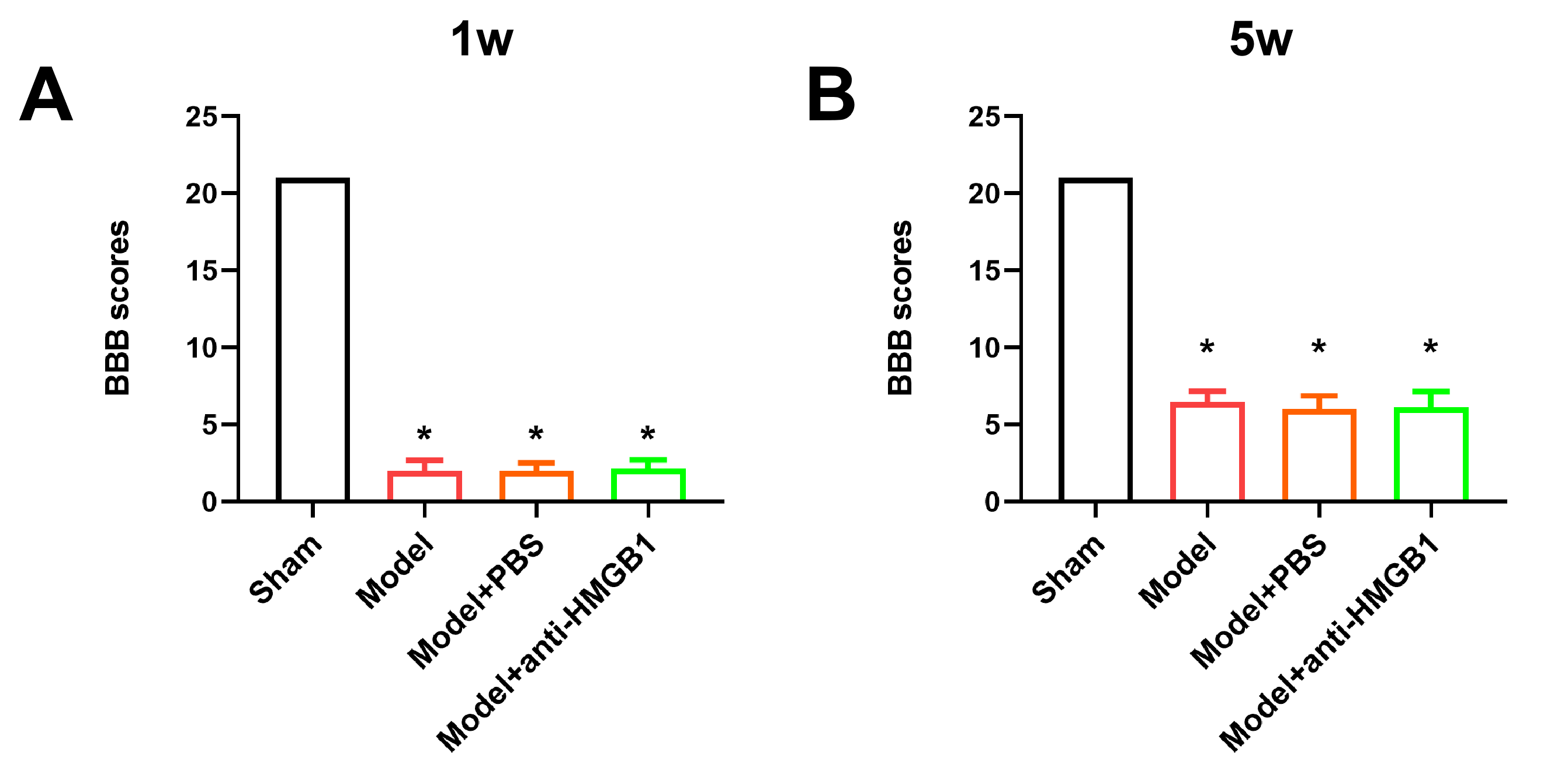


**Fig. S2**


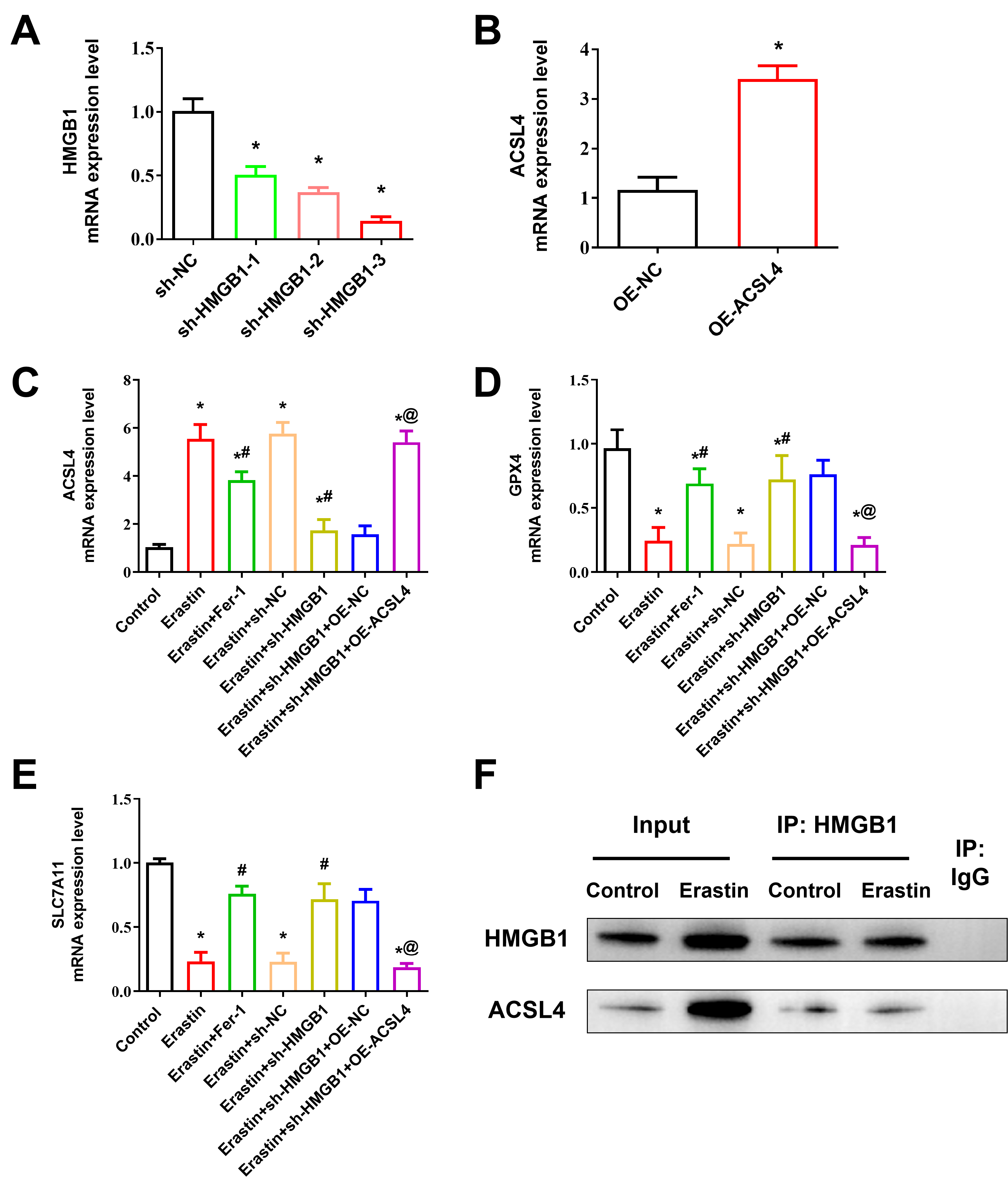

Supplement: Supplementary file 1 — Figure S1: The Basso, Beattie and Bresnahan (BBB) score results in different groups of rats. (A) 1 weeks post‐SCI (n = 12). (B) 5 weeks post‐SCI (n = 12). The data are presented as mean ± SD. *p < 0.05 versus Sham. Figure S2: HMGB1 regulates neuronal ferroptosis through targeted modulation of ACSL4. (A) Transfection efficiency of sh‐HMGB1s in neurons was verified by RT‐qPCR. (B) Transfection efficiency of ACSL4 overexpression plasmid in neurons was verified by RT‐qPCR. (C–E) The mRNA expression levels of ACSL4, GPX4 and SLC7A11 in neurons of different groups were detected by RT‐qPCR. (F) The CO‐IP experiment was performed to investigate the specific interaction between HMGB1 and ACSL4. [file JCMM-30-e71255-s001.docx]
