# Supplementary material for: Anti‐HMGB1 Antibody Therapy Ameliorates Depression Following Spinal Cord Injury in Rats by Inhibiting Ferroptosis
Source: J Cell Mol Med. 2026 Jun 23;30(12):e71255. doi: 10.1111/jcmm.71255 (PMC13290662; doi:10.1111/jcmm.71255)
Supplement: Supplementary file 2 — Table S1: The sucrose preference rate of rats at 4 weeks post‐SCI. [file JCMM-30-e71255-s002.docx]

**Supplementary Table 1**

The sucrose preference rate of rats at 4 weeks post-SCI

| Group | | A | B | C | D | E |
| --- | --- | --- | --- | --- | --- | --- |
| Sucrose Preference (%) | 1 | 90.85 | 59.55 | 55.12 | 57.88 | 59.28 |
|  | 2 | 91.11 | 56.66 | 56.78 | 58.42 | 60.88 |
|  | 3 | 90.02 | 57.14 | 57.34 | 59.67 | 61.17 |
|  | 4 | 93.35 | 58.21 | 58.91 | 60.19 | 62.49 |
|  | 5 | 95.13 | 56.69 | 59.03 | 61.54 | 63.01 |
|  | 6 | 89.88 | 58.84 | 60.45 | 62.33 | 64.55 |
|  | 7 | 88.79 | 59.21 | 61.22 | 63.77 | 55.89 |
|  | 8 | 92.23 | 56.21 | 62.67 | 64.02, | 56.61 |
|  | 9 | 89.56 | 56.31 | 63.89 | 55.31 | 57.72 |
|  | 10 | 87.98 | 60.04 | 64.11 | 56.94 | 58.06 |
|  | 11 | 91.12 | 62.34 | 55.76 | 57.05 | 59.44 |
|  | 12 | 90.02 | 63.33 | 56.23 | 58.73 | 60.77 |

Group A: Control; Group B: Model; Group C: Model+PBS; Group D: Model+anti-IgG; Group E: Model+anti-HMGB1.
